# Shear bond strength of ceramic laminate veneers to finishing surfaces with different percentages of preserved enamel under a digital guided method

**DOI:** 10.1186/s12903-021-02038-5

**Published:** 2022-01-07

**Authors:** Jiakang Zhu, Jing Gao, Luming Jia, Xin Tan, Chenyang Xie, Haiyang Yu

**Affiliations:** 1 State Key Laboratory of Oral Diseases, Department of Prosthodontics, National Clinical Research Center for Oral Diseases, West China Hospital of Stomatology, Sichuan University, 14 Renmin South Road, 3rd section, Chengdu, 610041 Sichuan People’s Republic of China; 2 BYBO Dental Hospital, Beijing, People’s Republic of China; 3 Department of Dental Technology, West China Hospital of Stomatology, Sichuan University, Chengdu, People’s Republic of China

**Keywords:** Ceramic laminate veneers, Enamel preservation, Shear bond strength, 3D-printed guide, Finishing surface

## Abstract

**Background:**

The purpose of this in vitro study was to evaluate the effect of the percentages of preserved enamel on ceramic laminate veneers’ (CLVs) shear bond strength (SBS).

**Methods:**

Seventy extracted human maxillary central incisors were scanned and reconstructed into three-dimensional models. The extracted teeth were then embedded and randomly divided into seven groups (n = 10 per group). Based on digital analyses of the three-dimensional models, guided tooth preparation and bonding procedures were performed individually to form seven different percentages (100%, 80%, 60% 50%, 40%, 20% and 0%) of remaining enamel thickness on the bonding surface. Finally, the SBS test was performed, and the data were statistically analysed by one-way ANOVA with LSD post hoc test (α = 0.05).

**Results:**

The complete enamel surface exhibited the highest SBS (19.93 ± 4.55 MPa), followed by 80% enamel (19.03 ± 3.66 MPa), 60% enamel (18.44 ± 3.65 MPa), 50% enamel (18.18 ± 3.41 MPa), 40% enamel (17.83 ± 3.01 MPa) and 20% enamel (11.32 ± 3.42 MPa) group. The lowest SBS (9.63 ± 3.46 MPa) was detected in 0% enamel group. No significant difference was observed among the 40–100% enamel groups, while the 20% or 0% enamel group demonstrated a significantly lower mean SBS than the 40% enamel group (*p* < 0.05).

**Conclusion:**

The SBS value of CLVs bonded to 100% enamel on the finishing surfaces (nearly 20 MPa) was twice that which bonded to 0% enamel (nearly 10 MPa). Bonding to 100% enamel is the most reliable treatment. When dentin exposure is inevitable, enamel should be preserved as much as possible to maintain good bonding. In addition, 40% of preserved enamel on the bonding surface was the minimal acceptable value to fulfil the requirements of good bonding strength.

## Background

Laminate veneers are clinically indicated for various aesthetic reasons, leading to a more minimally invasive treatment by allowing more tooth structure to be preserved [1]. However, laminate veneers are also prone to failure because of the higher technical sensitivity [2, 3]. Debonding is one of the most common reasons for the failure of laminate veneers, and there are many factors contributing to debonding, such as the types of adhesive system and resin cement, tooth preparation depths and types, and functional and parafunctional activities, etc. [4, 5] As one of the most important factors affecting debonding, the depths of preparation influence the percentage of enamel surface on the adhesive surfaces, which is crucial for maintaining enough bonding strength of laminate veneers. Previous in vitro studies have confirmed that the shear bond strength (SBS) of ceramic to enamel was higher than to dentin [6, 7], and longitudinal studies have shown that the survival rate of laminate veneers after 10 years is more than 90% if the enamel bonding surface is sufficient [8, 9]. However, the effects of enamel preservation on the shear bond strength (SBS) of laminate veneers have not been clearly quantified.

Computer-guided tooth preparation is developed from the technologies of dental scanners, computer-aided design (CAD) software, and computer-aided manufacturing (CAM) software. Guided tooth preparation promotes precisely controlled depths of preparation, and digital spatial analysis facilitates the completion intra-enamel preparation [10–12]. Nevertheless, in regard to discoloured or misaligned teeth, more reduction of tooth structure may be required to improve the aesthetic result, causing inevitable dentin exposure [13]. For laminate veneers, what is the acceptable range of dentin exposure? Öztürk and others [6] indicated that compared with bonding to enamel, the bonding strength of ceramic to enamel–dentine complex had no significant reduction. However, it is worth noting that the enamel/dentin ratio was estimated to be approximately 1:1 in that study without accurate calculation. Some dentin exposure was allowed in the treatment [14], but further research about the critical value was deficient.

The aims of this study were to create different percentages of preserved enamel amount on finishing surfaces through a digital guided method and to compare the SBS of the different percentages of preserved enamel. In addition, it is expected to preliminarily explore the acceptable extent of dentin exposure for CLVs. The null hypothesis was that there is no association between the percentages of preserved enamel amount and SBS values for CLVs on maxillary central incisors.

## Methods

### Specimen collection

**Fig. 1:**
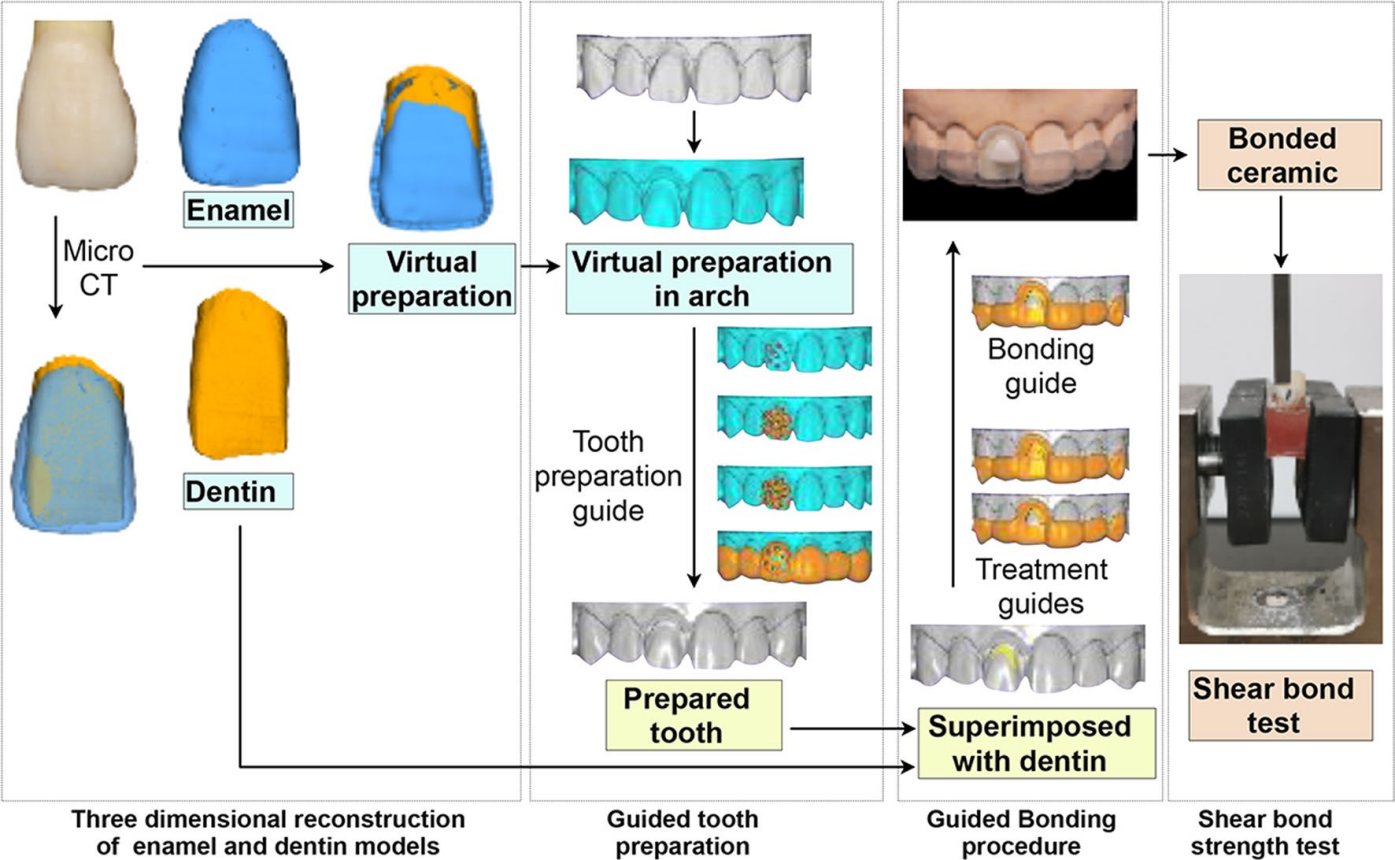
The workflow diagram of this study

The protocol of this study was approved by the Ethics Committee (Approval Number: WCHSIRB-D-2019-122). Seventy non-carious human maxillary central incisors were stored in 0.5% chloramine in water at 4 °C and used within 1 month after extraction. Figure 1 illustrates the workflow diagram of this study.

### Design of tooth preparation guides

The extracted teeth were scanned by micro-computed tomography (micro-CT) (μCT 50; SCANCO Medical AG, Bassersdorf, Switzerland) (scanning parameters: 80 kV, 500 μA, 19.64 μm, and 800 ms) to reconstruct their three-dimensional (3D) enamel and dentin models in reverse engineering software (Mimics 17.0; Materialise, Leuven, Belgium). All teeth were mounted in dental gypsum to simulate the dental arch, which was scanned by an intraoral scanner (TRIOS Colour Pod; 3Shape, Copenhagen, Denmark) and saved in a standard tessellation language (STL) format. The arch models were randomly divided into seven groups (n = 10 each). The analysis based on the power.anova.test function of the stats package (3.6.2) showed that using 10 specimens per group would give a needed power. And this is also consistent with the number of specimens used in the recently published literatures on shear bond strength test [15–17].

Each digital arch model was imported into Materialise software (Magics 23; Materialise, Leuven, Belgium), and virtual preparation was conducted on the sample tooth, as described by Gao et al. [10]. Briefly, the labial surface was selected and shifted inward by using the “Offset” tool. Different depths of preparation were designed to form different percentages of preserved enamel on the finishing surfaces in seven groups. To convert the depths of virtual preparation to definitive preparation, tooth preparation guides were designed based on virtual preparation, as described by Liu et al. [12].

### Guided tooth preparation

**Fig. 2:**
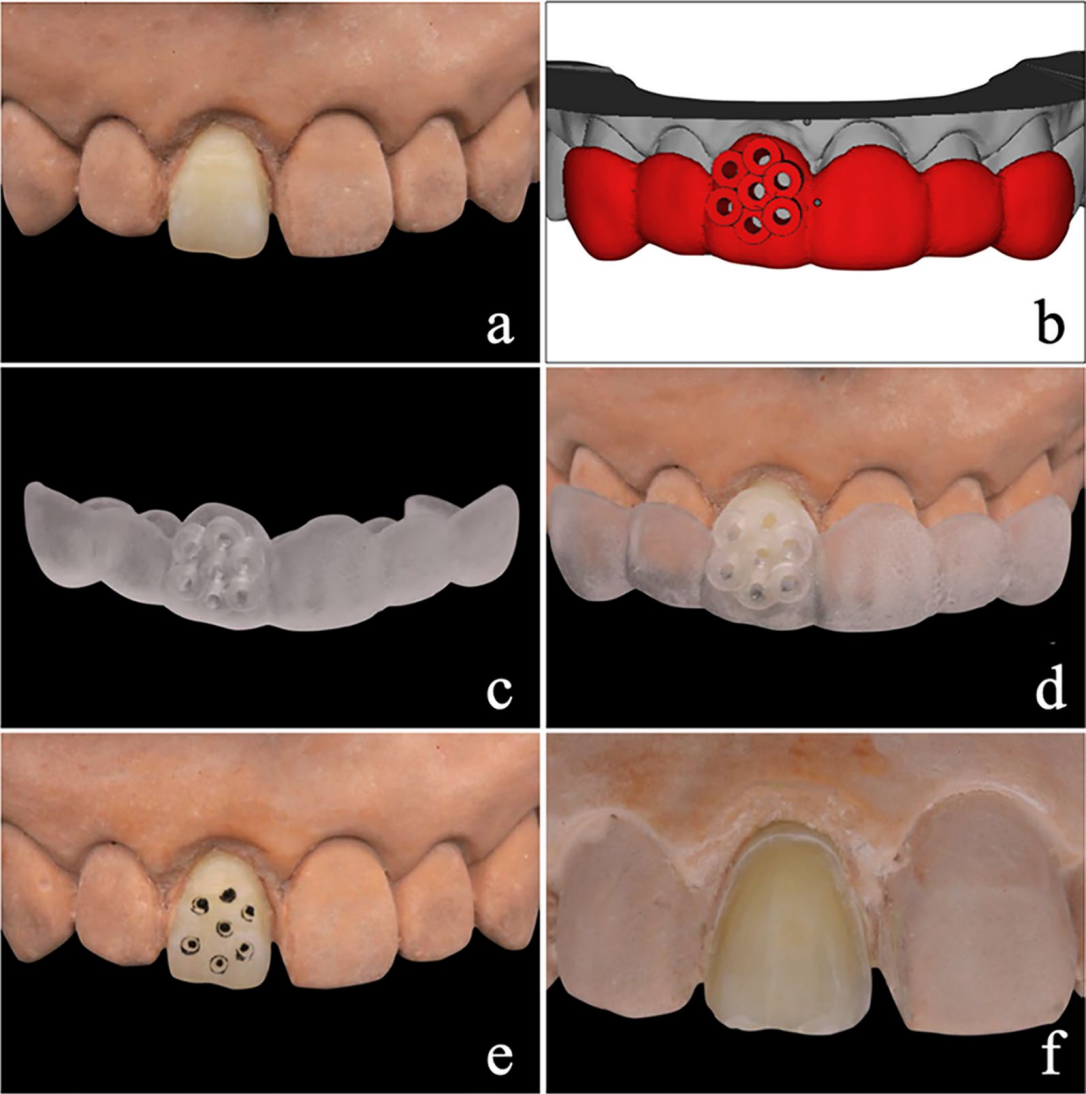
Guided tooth preparation. **a** Embedded tooth. **b** Design of tooth preparation guide. **c** Tooth preparation guide. **d** Location of tooth preparation guide. **e** Depth control holes. **f** Prepared tooth surface

Guided tooth preparation was conducted by one operator who was unaware of the experimental aims and assessment criteria of the study (Fig. 2). Briefly, depth-guiding dimples were created with the aid of a tooth preparation guide by using a calibrated bur (HX-1; Gaofeng, Wuxi, China). Then, the dimples were marked with a pencil, the remaining tooth tissues between the dimples were removed and tooth surfaces were finished by a tapered carbide bur (HX-2; Gaofeng, Wuxi, China). Following this process, the depth of preparation was consistent with that of the virtual preparation, which was designed to form a specific enamel/dentin ratio on the tooth surface. The prepared teeth were scanned and saved as “STL” files, and the specimens were stored in artificial saliva.

### Fabrication of ceramic laminate veneers

**Fig. 3:**
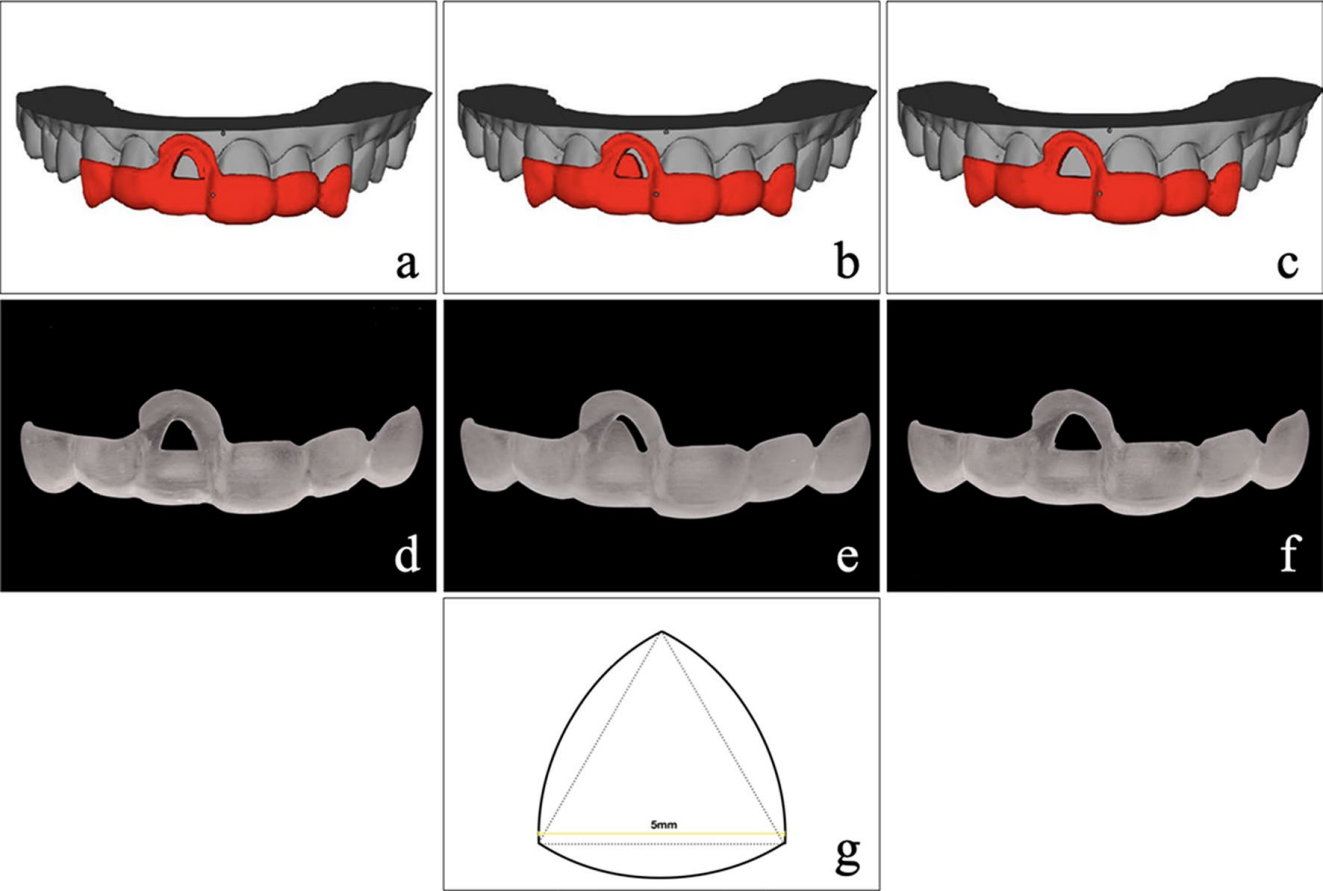
Adhesive guides. **a** Design of enamel treatment guide. **b** Design of dentine treatment guide. **c** Design of bonding guide. **d** Enamel treatment guide. **e** Dentine treatment guide. **f** Bonding guide. **g** The form of adhesive surface: a reuleaux triangle

Lithium disilicate ceramic mini veneers (IPS e. max Press; Ivoclar Vivadent, Schaan, Liechtenstein) were designed and milled by a chairside CAD/CAM machine (Cameo N4; Aidite, Qinhuangdao, China). To simulate the shape of the veneer, the adhesive surface was in the form of a Reuleaux triangle whose sides were replaced with circular arcs with a curve of constant width (Fig. 3g). The adhesive surfaces of all the specimens were finished with 600-grit abrasive paper to create a uniform surface. All specimens were cleaned with 75% alcohol, washed in deionized water by ultrasonication for 5 min, and dried at room temperature.

### Design of the treatment and adhesive guides

The digitally prepared tooth was superimposed with its enamel and dentin models in dental CAM software (Exocad 2018; Exocad GmbH, Darmstadt, Germany), where the enamel and dentin surfaces were presented on the tooth surface. Then, the same Reuleaux triangle was shifted on to the finishing surface until the percentage of preserved enamel in the area of the Reuleaux triangle fulfilled the requirement. Thus, the adhesive surface of the tooth, as the shape of the veneer, was designed to form a respective percentage of preserved enamel in each group (Table 1). Accordingly, the treatment guides were designed to locate the enamel and dentin surfaces, and the bonding guides were designed to locate the adhesive surface (Fig. 3). All guides were fabricated using the 3D printer (ProJet MJP 3600; 3D Systems, Rock Hill, SC).

**Table 1:** The percentages of preserved enamel on the selected adhesive surface in seven gropes

Groups	G1	G2	G3	G4	G5	G6	G7
The percentages of preserved enamel	100	80	60	50	40	20	0

### Guided bonding procedure

**Fig. 4:**
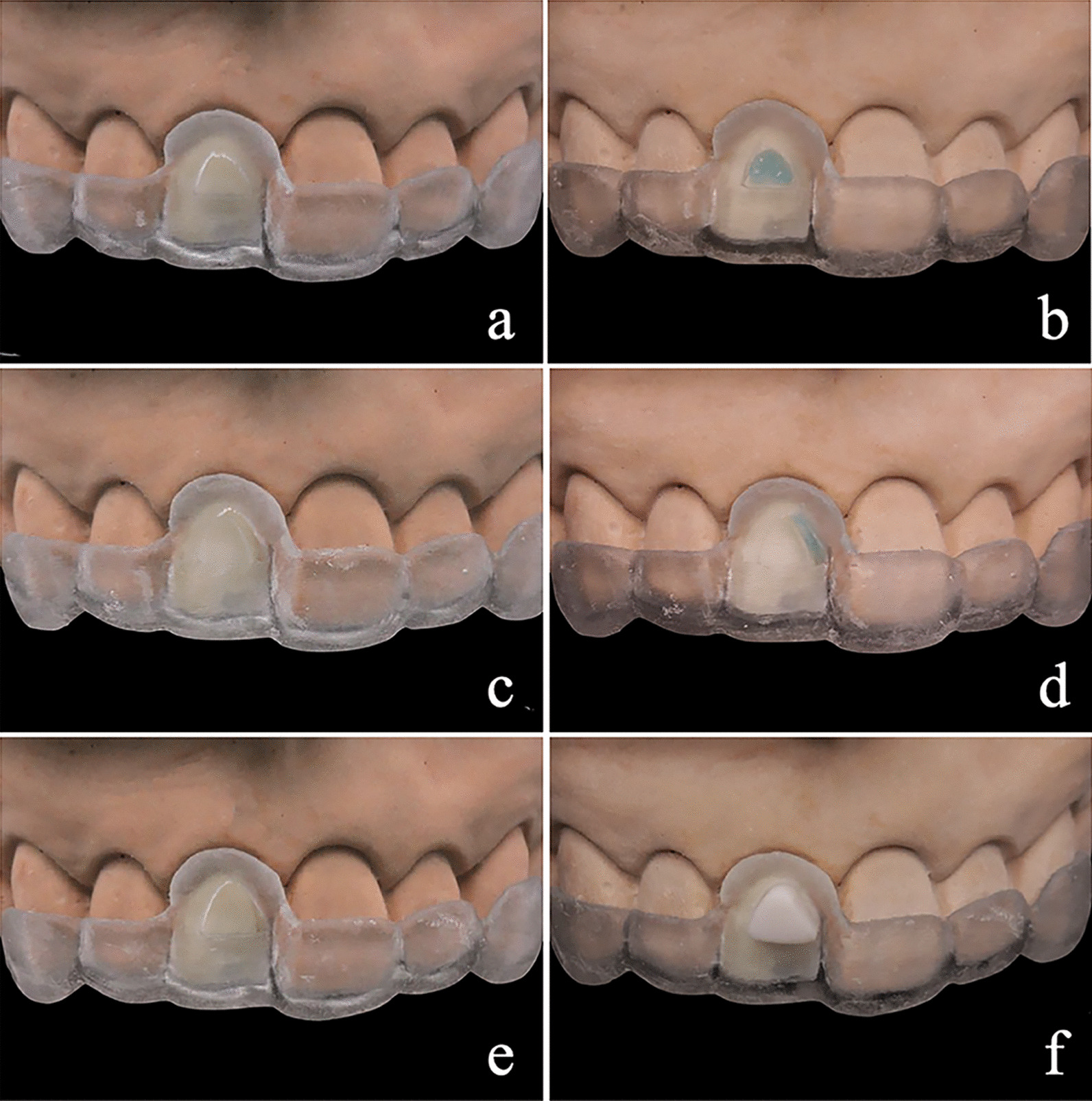
Guided bonding procedure. **a** Location of enamel treatment guide. **b** Enamel treatment under guide with 37% H_3_PO_4_ for 30 s. **c** Location of dentine treatment guide. **d** Dentine treatment under guide with 37% H_3_PO_4_ for 3 s. **e** Adhesive surface treatment under bonding guide with single bond for 10 s. **f** Ceramic bonded on the tooth

Figure 4 shows the bonding procedure in the present study. Enamel and dentin surfaces were treated under the treatment guides following the treatment procedures in Table 2. Then, the bonding guide was seated, and adhesion was performed according to the instructions of the manufacturers (Rely X veneer; 3 M, Sao Paulo, MN). Finally, the bonding guide was cut with a tapered fissure bur and gently removed. A pressure control (100 N) was conducted to standardize the bonding procedure and to withstand the debonding forces from the removal of the guide.

**Table 2:** Surface procedures of the teeth and ceramics

Surface	Surface treatments
Porcelain	5% HF for 60 s; primer for 60 s
Enamel	37% H_3_PO_4_ for 30 s; single bond for 10 s
Dentin	37% H_3_PO_4_ for 3 s; single bond for 10 s

H3PO4: phosphoric acid (total etch, ivoclar vivadent, schaan, liechtenstein). HF: hydroflfluoric acid (vita ceramics etch, VITA zahnfabrik, Bad Sackingen, Germany)

### The SBS test

The sample tooth was removed from the arch and vertically embedded in acrylic resin (polymethyl methacrylate; Nissin, Tokyo, Japan) to place the cementoenamel junction (CEJ) plane under 2.5 mm ± 0.5 mm from the top platform of the resin surface. The specimens were stored in artificial saliva for 24 h at 37 °C and were thermocycled between 5 and 55 °C in deionized water for 5000 cycles. The test head was sheared from incisal to cervical. The SBS test was conducted with a universal mechanical testing machine (INSTRON-5565; INSTRON, Boston, MA) at a crosshead speed of 0.5 mm/s until failure on all samples. The test results were recorded in MPa.

### Failure analysis and scanning electron microscopy (SEM) examination

After the SBS test, all specimens were examined under a stereomicroscope (BX51M; OLYMPUS, Tokyo, Japan) at a magnification of 50 × to determine the fracture mode. Representative specimens were selected for fractographic examination by SEM (Inspect F50; FEI, Hillsboro, OR) at 5000 × magnification. Possible failure modes that were similar to the classification by Scherrer and others [18] and Öztürk and others [6] were classified as follows:

1. Adhesive failure: fracture between the ceramic and tooth surface within the adhesive interface,
2. Mixed failure: are cohesive failures (less than 40% of the interface) in the tooth structure and adhesive interface combined with adhesive failure between the ceramic and tooth,
3. Cohesive failure: internal fracture of the tooth structure or adhesive interface (more than 40% of the bonding interface).
4. To quantify the proportion of internal fractures, Image J software was used to estimate the percentage of internal fracture area.

### Statistical analysis

Numerical (quantitative) data were presented as the mean and standard deviation values. Categorical (qualitative) data were presented as frequencies and percentages. One-way analysis of variance and the least significant difference (LSD) test were used to calculate statistics on the fracture strength among different groups. Fisher's exact test was used to compare the failure modes of the four groups. The test standard was bilateral α = 0.05. The significance level was set at *p* < 0.05. Statistical analysis was performed with SPSS software (SPSS 26.0; SPSS, Chicago, IL).

## Results

**Fig. 5:**
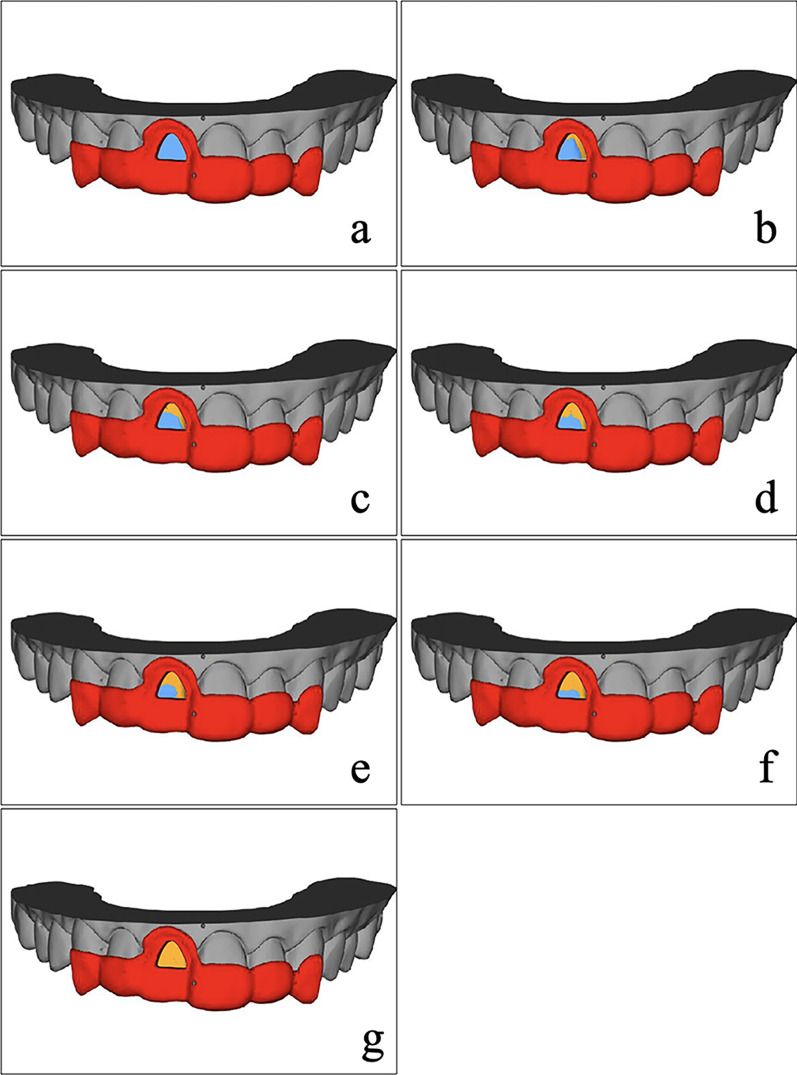
The adhesive surfaces from seven groups. **a** 100% enamel presented. **b** 80% enamel presented. **c** 60% enamel presented, **d** 50% enamel presented, **e** 40% enamel presented. **f** 20% enamel presented, **g** 0% enamel presented

Figure 5 shows the selected adhesive surfaces from different groups. The finishing surfaces were reconstructed by merging the 3D dentin model and the prepared tooth model. As shown in Fig. 5a–g, the percentages of preserved enamel amount on selected adhesive surfaces of the seven groups were 100%, 80%, 60%, 50%, 40%, 20% and 0%.

**Fig. 6:**
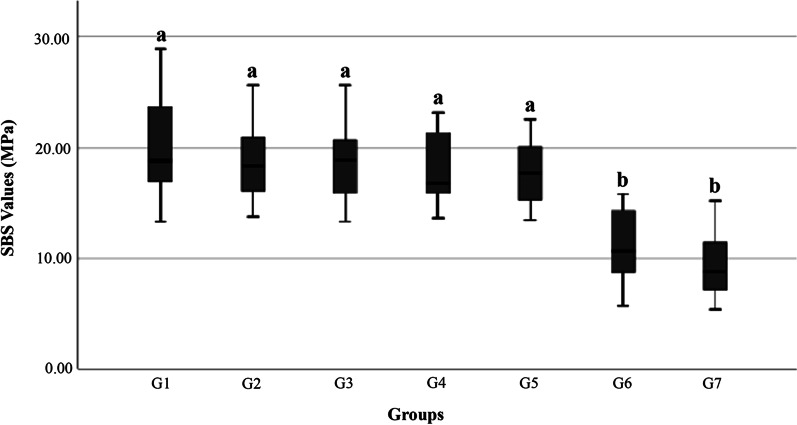
The shear bond strength test in seven groups. Different lower-case letters indicate the significant statistical difference (*p* < 0.05)

Figure 6 presents the SBS values of different percentages of preserved enamel. Among the 7 groups, G1 exhibited the highest mean SBS value (19.93 ± 4.55 MPa), followed by G2 (19.03 ± 3.66 MPa), G3 (18.44 ± 3.65 MPa), G4 (18.18 ± 3.41 MPa) and G5(17.83 ± 3.01 MPa) groups. No significant difference was observed among the G1, G2, G3, G4 and G5 groups (*p* > 0.05), but G5 demonstrated a significantly higher SBS value thanG6 (11.32 ± 3.42 MPa) (*p* < 0.05). G7 showed the lowest mean SBS value (9.63 ± 3.46 MPa) among these groups, but there was no significant difference between the G6 and G7 groups (*p* > 0.05).

**Fig. 7:**
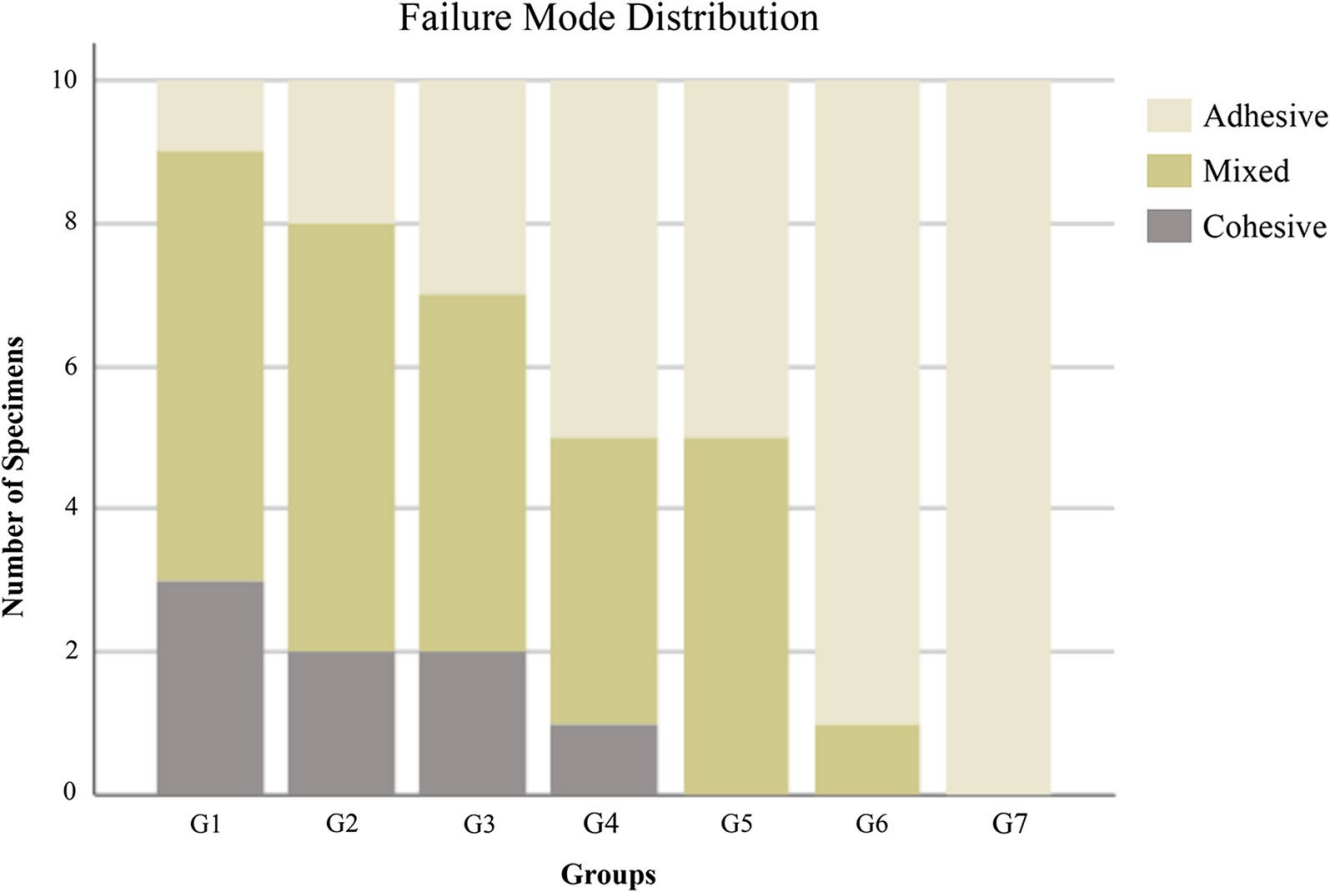
Distribution of adhesive failure types among seven groups

The failure mode distribution in the seven groups is presented in Fig. 7. The most frequently experienced failure type was adhesive failures in all groups, and the G7 group was the most frequent, with 10 failures (100%). Mixed failures were observed in 6 groups except for G7, among which the G1 and G2 groups had the most failures with 6 (60%). Cohesive failure was found in the G1, G2, G3 and G4 groups; among them, the G1 group had the greatest number (3; 30%).

**Fig. 8:**
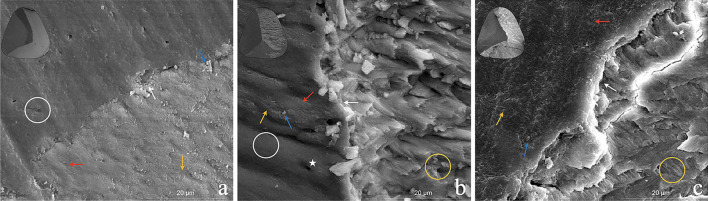
SEM micrographs of fractured specimens. **a** Cohesive failure from the G4 group. **b** Mixed failure from the G4 group. **c** Adhesive failure from the G4 group. white circle: dentin; yellow circle: enamel; yellow arrow: residual adhesive; White arrow: fracture interface; Red arrow: Internal structure of adhesive; blue arrow: filler content of adhesive; × 50 fracture interface in black circle at upper left corner

Figure 8 shows SEM micrographs of the tooth surfaces after the shear bond strength test. Figure 8a illustrates the cohesive failure of a specimen from the G4 group. This type of failure indicates that internal fracture of the tooth structure or adhesive interface occurred and the area was more than 40% of the bonding interface. Figure 8b is an example of a mixed failure from the G5 group. Mixed failure indicates a mixture of cohesive failure (less than 40% of the interface) and adhesive failure within the same fracture surface. Figure 8c shows representative micrographs of adhesive failure. The fracture occurred in the tooth structure or adhesive interface.

## Discussion

This study was the first to achieve quantitative classification of the percentages of preserved enamel amount on the finishing surface through a digital guided method. Tooth preparation guides were designed to transfer the reduction depths of virtual preparations to definitive preparation [19–21]. Enamel substrate was presented by the superimposition of the prepared tooth and its dentin models [22, 23], by which the adhesive guides could be designed to locate the enamel and dentin adhesive surface. With the aid of the surface treatment guides, the enamel and dentin surfaces could be presented and treated separately without impacting each other. The adhesive surface of mini veneers was designed as a Reuleaux triangle to simulate the shape of veneers in the clinic, based on a method comparable to the ISO 29022 shear test [24]. With the help of the digital workflow, seven levels of the percentages of preserved enamel amount on the finishing surface were established and precisely transferred to the teeth specimens.

In this study, the percentages of preserved enamel amount had a significant effect on the shear bond strength, and the results rejected the null hypothesis that there was no association between the percentages of preserved enamel and SBS values for CLVs on maxillary central incisors. In this research, we discovered that the 100% enamel surface group showed the highest mean SBS value among the 7 groups, which correlated with previous reports [6]. The SBS value of CLVs bonded to 100% enamel on the finishing surfaces was nearly 20 MPa, which was twice that bonded to dentin. The results indicated that bonding to complete enamel is still the most reliable treatment. The degree of mineralization in enamel is higher than that in dentin. The honeycomb structure formed after demineralization of hydroxyapatite is favourable for the formation of resin protrusion in enamel. However, there are more organic components in dentin, and dentin tubules contain much water. Inappropriate acid-etching and drying can lead to the collapse of the collagen fibre network, which has a great impact on dentin bonding [25, 26]. Collectively, tooth preparation for CLVs should be controlled in enamel as much as possible to ensure the highest SBS value.

The study showed that there was no significant difference in bond strength among the 40–100% enamel groups, indicating that a small amount of dentin exposure is acceptable during preparation for CLVs in clinics. We also observed that there was no significant difference in bond strength between the 20% and 0% enamel groups. Thus, extensive dentine exposure should be avoided. Notably, bonding to 40% enamel demonstrated significantly higher bond strength than bonding to 20% enamel. Previous studies have reported that more debonding of CLVs would be presented when less than 50% of enamel was preserved [13, 27, 28]. However, the value (50%) was just a ballpark estimate, lacking relatively accurate calculation. It has been reported that the SBS value of adhesives to dentin should be at least 17 MPa, while that of enamel should be 20 MPa to adequately compensate for the stresses caused by polymerization shrinkage [6, 29]. Owing to the different experimental conditions and methods, in this study, the mean shear bond strength values of the 40–100% enamel groups were above 17 MPa. However, mean shear bond strength values of 20% and 0% groups were well below 17 MPa. Considering all these data and our results, it is reasonable to suggest that CLVs could be applied only when at least 40% of enamel is preserved on the finishing surface after preparation to guarantee good bonding.

In addition, cohesive failure is only observed in bonding to 50–100% enamel groups. Cohesive failure is attributed to the adhesive bond strength exceeding the intrinsic strength of the tooth, so this type of failure indicates increased bond strength between the resin cement and teeth [30, 31]. This is consistent with the higher SBS values in these four groups. However, structural features of the extracted teeth, increased fragility of teeth after a long storage time, and nonuniform distribution of stress, are also predisposing factors for cohesive failures [32]. Adhesive failure was the most frequent fracture type, bonding to 20% enamel presented significantly more adhesive failures than 40% enamel, and bonding to 0% enamel presented only adhesive failures. This result is in accordance with previous investigations that showed that when the shear bond strength values of resin cement to the dentin surface are lower, adhesive bond failure is more likely to occur [33, 34]. Taken together, these results confirmed the critical role of 40% enamel preservation in tooth preparation for CLVs, which demonstrates significantly higher bond strength than bonding to 20% enamel.

Due to the limitation of the SBS test, the specimen of CLVs was designed as a mini veneer like Reuleaux triangle, which could not completely simulate the actual clinical situation. In addition, experimental conditions were different from clinical treatment. However, the study still had certain guiding significance for clinicians. For CLVs, tooth preparation should be finished in enamel as far as possible to reduce the risk of failure. In regard to inevitable dentin exposure [35], the percentages of enamel adhesive surfaces should be more than 40% to guarantee the longevity of CLVs, otherwise, other treatments such as full crowns, may be considered [36, 37]. In addition, it has been suggested that the preparation margins should be in sound enamel for CLVs to improve the bonding strength between teeth and restorations as well as reduce the incidence of secondary caries caused by microleakage [38, 39].

## Conclusions

Within the limitations of this study, the following conclusions can be addressed:

1. Complete intra-enamel preparation is the most optimal for CLVs by providing the highest SBS values. Enamel preservation of 40% is the essential threshold value during tooth preparation for CLVs to ensure enough bond strength of CLVs.
2. The digital guided method promotes a more reliable process for the enamel preservation. Tooth preparation guide improves the accuracy of reduction depth for veneer preparation, and adhesive guides allow the location of enamel, and dentin on the adhesive surfaces.

## Data Availability

The data of this study are available from corresponding author on reasonable request.

## Abbreviations

CLVs: Ceramic laminate veneers
SBS: Shear bond strength
CAD: Computer-aided design
CAM: Computer-aided manufacturing
3D: Three-dimensional
STL: Standard tessellation language
CEJ: Cemento-enamel junction
SEM: Scanning electron microscopy
